# Near-Complete Genome Sequence of a Representative Strain within a Rare Foot-and-Mouth Disease Virus O/ME-SA/Ind2001BD2 Sublineage from Bangladesh

**DOI:** 10.1128/MRA.00705-19

**Published:** 2019-08-01

**Authors:** A. S. M. Rubayet Ul Alam, M. Rahmat Ali, Mohammad Anwar Siddique, Huzzat Ullah, Munawar Sultana, M. Anwar Hossain

**Affiliations:** a Department of Microbiology, University of Dhaka, Dhaka, Bangladesh; University of Rochester School of Medicine and Dentistry

## Abstract

The near-complete genome sequence of a foot-and-mouth disease virus (FMDV), strain O/ME-SA/Ind2001BD2, isolated exclusively from Bangladesh, is reported here. Amino acid substitutions at critical antigenic sites of the capsid were identified compared to the surface proteins of existing vaccine strain O/India/R2/75 and contemporary FMDV serotype O isolates of Bangladesh.

## ANNOUNCEMENT

Foot-and-mouth disease virus (FMDV) is an RNA virus belonging to the Aphthovirus genus in the *Picornaviridae* family with various topotypes, lineages/groups, and sublineages under seven serotypes, O, A, C, Asia1, SAT1, SAT2, and SAT3 (1). In Bangladesh, serotypes O, A, and Asia1 cause the FMD outbreaks, and recently emerged novel sublineages (Ind2001BD1 and Ind2001BD2) along with previously circulating sublineage Ind2001d strains within the O/ME-SA/Ind2001 lineage were reported (2–4). Previously, we reported the complete genome sequences of those three circulatory serotypes and two representative isolates of the Ind2001d (BAN/NA/Ha-156/2013) and Ind2001BD1 [BAN/GO/Ka-236(Pig)/2015] sublineages, respectively (5–8).

Here, we report the first near-complete genome sequence of a rare virus strain (BAN/BO/Na-161/2013) within the novel Ind2001BD2 sublineage collected from the tongue epithelium of an infected cow at Nandigram, Bogra, Bangladesh, on 7 July 2013. The virus was isolated using the BHK-21 cell line, and viral RNA was extracted from infected cell culture supernatant of passage 3 in an automated Maxwell 16 system using the Maxwell 16 total RNA purification kit (Promega, USA), followed by cDNA synthesis using the GoScript reverse transcription system with oligo(dT) and random primers. Afterwards, 17 overlapping FMDV-specific primer pairs based on the BAN/NA/HA-156/2013 sequence were used to amplify the near-complete genome (6, 7). The amplicons were sequenced on an ABI genetic analyzer using Z-BigDye Terminator kit v3.0 (Applied Biosystems, USA) and assembled into a near-complete genome using SeqMan version 7.0 (DNAStar Lasergene, USA) with default settings. Finally, we performed ClustalW algorithm-based codon alignment, using default parameters, followed by mutational analysis and reconstruction of the phylogenetic tree using the maximum likelihood method based on the Tamura-Nei parameter model in MEGA7 (9).

**FIG 1:**
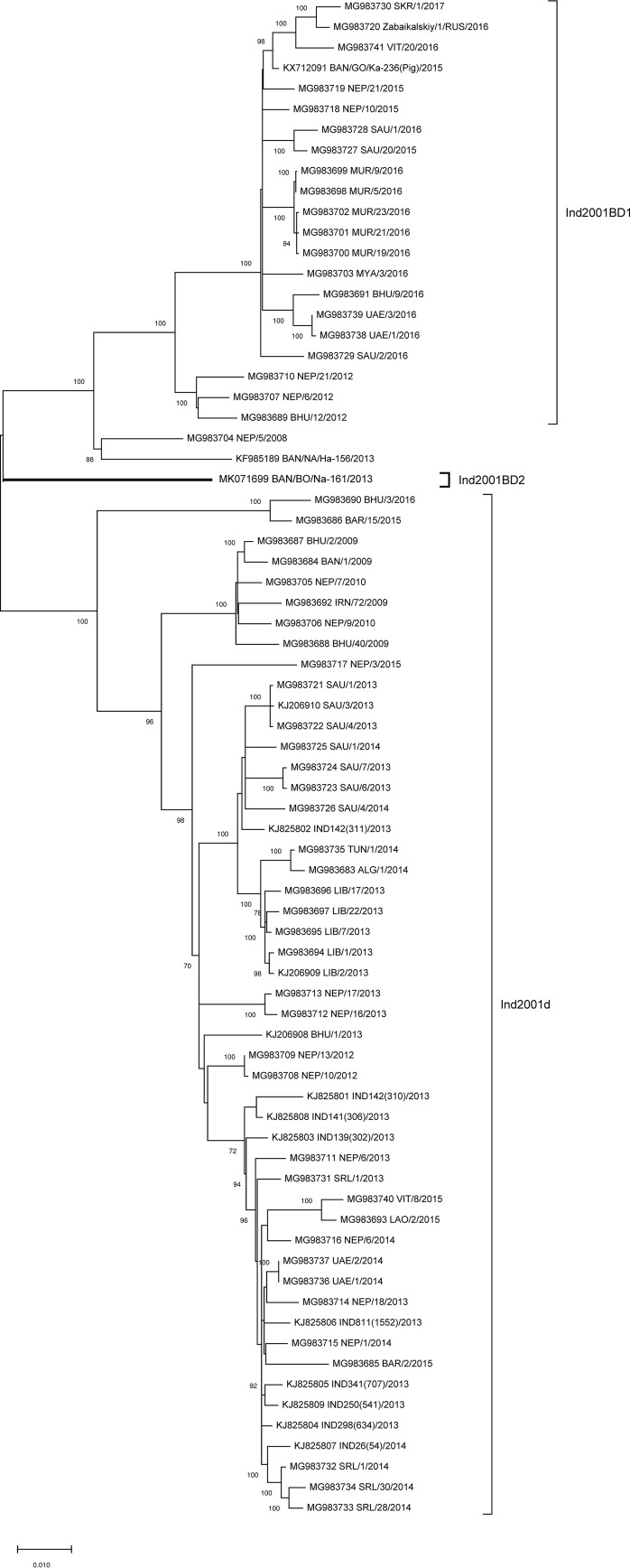
Phylogenetic tree reconstructed based on entire coding sequence (6,996 nucleotides) of 74 virus strains from three sublineages of the Ind2001 lineage using the maximum likelihood method and Tamura-Nei model in addition to a discrete gamma distribution (+G, parameter = 1.2961) and evolutionarily invariable ([+I], 36.30% sites) rate variation model with 1,000 bootstrap replicates. The only isolate of the Ind2001BD2 sublineage is shown in a larger font. The other isolates are of sublineages, Ind2001BD1 and Ind2001d, which are also found circulatory in Bangladesh as well as neighboring countries.

The near-complete genome is 8,221 nucleotides (nt) long with 53.76% GC content, including a 1,101-nt lengthy 5′ untranslated region (UTR) with a 16-nt poly(C) tract and a 3′ UTR of 221 nt trailed by an ≥23-nt poly(A) tail. The 6,999-nt-long open reading frame encodes 2,332 amino acids. The coding sequence (CDS)-based phylogeny (Fig. 1) corroborated the VP1 phylogeny (3), which clustered the virus into a clade that was distinct from those of circulatory isolates of different Ind2001 sublineages.

The maximum identities of the near-complete genomic nucleotide and the CDS-encoded amino acid sequence of BAN/BO/Na-161/2013 were determined at 94% and 99%, respectively, with other virus strains from Bangladesh, Bhutan, and Nepal. Compared to the vaccine strain (O/India/R2/75, GenBank accession number AF204276), BAN/BO/Na-161/2013 capsid proteins (VP1 to VP3) had nine adaptive mutations (D138E, S140A, I144V, N197S, E198T, L212F, A70V, N134K, and G60D) at three antigenic sites (1, 2, and 4, respectively). Also, BAN/BO/Na-161/2013 contained five amino acid substitutions (T156A, Q198T, L212F, P74S, and S133Q) in the VP1 and VP2 antigenic regions in comparison with BAN/NA/Ha-156/2013, a contemporary circulatory virus in Bangladesh, predicted as a potential vaccine candidate earlier (7).

Our study presents the near-complete genome sequence of a unique virus strain of the Ind2001BD2 sublineage identified only in Bangladesh, which might pose a threat to vaccine escape since it contains substantial changes in the capsid region.

### Data availability.

The near-complete genome sequence of BAN/BO/Na-161/2013 and the reads have been published in GenBank and the Sequence Read Archive under the accession numbers MK071699 (GenBank) and SRX5914390 to SRX5914422 (SRA).

## References

[1] Brito BP, Rodriguez LL, Hammond JM, Pinto J, Perez AM 2017 Review of the global distribution of foot-and-mouth disease virus from 2007 to 2014. Transbound Emerg Dis 64:316–332. doi:10.1111/tbed.12373. 25996568

[2] Nandi SP, Rahman MZ, Momtaz S, Sultana M, Hossain MA 2015 Emergence and distribution of foot-and-mouth disease virus serotype A and O in Bangladesh. Transbound Emerg Dis 62:328–331. doi:10.1111/tbed.12113. 23734722

[3] Siddique MA, Ali MR, Alam A, Ullah H, Rahman A, Chakrabarty RP, Amin MA, Hoque SA, Nandi SP, Sultana M, Hossain MA 2018 Emergence of two novel sublineages Ind2001BD1 and Ind2001BD2 of foot-and-mouth disease virus serotype O in Bangladesh. Transbound Emerg Dis 65:1009–1023. doi:10.1111/tbed.12834. 29457368

[4] Ullah H, Siddique MA, Al Amin M, Das BC, Sultana M, Hossain MA 2015 Re-emergence of circulatory foot-and-mouth disease virus serotypes Asia1 in Bangladesh and VP1 protein heterogeneity with vaccine strain IND 63/72. Lett Appl Microbiol 60:168–173. doi:10.1111/lam.12354. 25370946

[5] Ali MR, Alam A, Amin MA, Ullah H, Siddique MA, Momtaz S, Sultana M, Hossain MA 2017 Complete genome sequence of the circulatory foot-and-mouth disease virus serotype Asia1 in Bangladesh. Genome Announc 5:e01135-17. doi:10.1128/genomeA.01135-17. 29074654 PMC5658492

[6] Ali MR, Ullah H, Siddique MA, Sultana M, Hossain MA 2016 Complete genome sequence of pig-originated foot-and-mouth disease virus serotype O from Bangladesh. Genome Announc 4:e01150-16. doi:10.1128/genomeA.01150-16. 27789636 PMC5084860

[7] Sultana M, Siddique MA, Momtaz S, Rahman A, Ullah H, Nandi SP, Hossain MA 2014 Complete genome sequence of foot-and-mouth disease virus serotype O isolated from Bangladesh. Genome Announc 2:e01253-13. doi:10.1128/genomeA.01253-13. PMC3916491 24503997

[8] Ullah H, Siddique MA, Sultana M, Hossain MA 2014 Complete genome sequence of foot-and-mouth disease virus type A circulating in Bangladesh. Genome Announc 2:e00506-14. doi:10.1128/genomeA.00506-14. 24926048 PMC4056291

[9] Kumar S, Stecher G, Tamura K 2016 MEGA7: Molecular Evolutionary Genetics Analysis version 7.0 for bigger datasets. Mol Biol Evol 33:1870–1874. doi:10.1093/molbev/msw054. 27004904 PMC8210823

